# Seed Size, Not Dispersal Syndrome, Determines Potential for Spread of Ricefield Weeds by Gulls

**DOI:** 10.3390/plants12071470

**Published:** 2023-03-27

**Authors:** Juan Manuel Peralta-Sánchez, Albán Ansotegui, Francisco Hortas, Stella Redón, Víctor Martín-Vélez, Andy J. Green, María J. Navarro-Ramos, Adam Lovas-Kiss, Marta I. Sánchez

**Affiliations:** 1 Departamento de Biología Vegetal y Ecología, Facultad de Biología, Universidad de Sevilla, Avda. Reina Mercedes 6, 41012 Seville, Spain; jmps@ugr.es (J.M.P.-S.); ansotegui.alban@gmail.com (A.A.); mredon@us.es (S.R.); 2 Departamento de Microbiología, Universidad de Granada, Avda. Fuentenueva s/n, 18071 Granada, Spain; 3 Wetland Ecology Department, Estación Biológica de Doñana, EBD-CSIC, Avda. Americo Vespucio 26, 41092 Seville, Spain; victormartin_velez@hotmail.com (V.M.-V.); ajgreen@ebd.csic.es (A.J.G.); mjnavarro@ebd.csic.es (M.J.N.-R.); 4 Instituto Universitario de Investigación Marina (INMAR), Campus de Excelencia Internacional del Mar (CEI·MAR), Universidad de Cádiz, Avda. República Árabe Saharaui s/n, 11510 Puerto Real, Spain; francisco.hortas@uca.es; 5 Wetland Ecology Research Group, Department of Tisza Research, MTA Centre for Ecological Research-DRI, H-4026 Debrecen, Hungary; lovas-kiss.adam@ecolres.hu

**Keywords:** endozoochory, lesser black-backed gull, *Larus fuscus*, dry-fruited seeds, fleshy-fruited seeds, dispersal syndromes

## Abstract

Recent field data suggest that migratory gulls disperse many rice field weeds by gut passage (endozoochory), most of which are dry fruited and widely assumed to have no long-distance dispersal mechanisms, except via human activity. We investigated this mechanism with a feeding experiment, in which seeds of five common rice field weeds (in order of increasing seed size: *Juncus bufonius*, *Cyperus difformis*, *Polypogon monspeliensis*, *Amaranthus retroflexus*, and the fleshy-fruited *Solanum nigrum*) were fed to seven individuals of lesser black-backed gulls *Larus fuscus* held in captivity. We quantified seed survival after collecting faeces at intervals for 33 h after ingestion, then extracting intact seeds and running germination tests, which were also conducted for control seeds. All five species showed high seed survival after gut passage, of >70%. Gut retention times averaged 2–4 h, but maxima exceeded 23 h for all species. Germinability after gut passage was 16–54%, and gut passage accelerated germination in *J. bufonius* and *S. nigrum*, but slowed it down in the other species. All species had lower germinability after gut passage compared to control seeds (likely due to stratification prior to the experiment), but the loss of germinability was higher in smaller seeds. There was no evidence that the different dispersal syndromes assigned to the five species (endozoochory, epizoochory or barochory) had any influence on our results. In contrast, mean gut retention time was strongly and positively related to seed size, likely because small seeds pass more quickly from the gizzard into the intestines. Non-classical endozoochory of dry-fruited seeds by waterbirds is a major but overlooked mechanism for potential long-distance dispersal, and more research into this process is likely essential for effective weed management.

## 1. Introduction

Seed dispersal by animals is a key process in the ecology and evolution of plants [1]. It affects recruitment, connectivity and gene flow among populations [2], with an important role in maintaining plant metacommunities [3,4]. Declines in seed dispersers can have cascading effects and impacts on plant populations, the composition and functional diversity of plant communities, the dynamics of multitrophic systems, and ecosystem functioning [5]. Animal-mediated seed dispersal is decisive for assuring the persistence and spread of plants under major drivers of biodiversity change such as habitat fragmentation, biological invasions and climate change [6,7].

Many plants can disperse their seeds by gut passage (endozoochory) inside birds or other animals. Major advances have been made in understanding seed dispersal of fleshy-fruited plants by frugivorous birds [8], but it is often wrongly assumed that only plants with fleshy fruits can be dispersed by endozoochory. This misunderstanding is likely perpetuated by the classical morphological dispersal syndromes applied to plant diaspores, which include a range of abiotic syndromes such as wind, water or gravity, and define an “endozoochory syndrome” based on the presence of a fleshy fruit [9]. However, non-classical endozoochory of plant seeds assigned to other syndromes is an important dispersal mechanism for many plant species [9,10]. For example, only 8% of hundreds of European plant species known to be dispersed by migratory ducks demonstrated endozoochory syndrome, and more research is needed into the internal dispersal of seeds by non-frugivorous birds [9,11]. Understanding the role of non-frugivorous birds in plant dispersal is vital to improve predictions about the consequences of global change, such as how plants respond to climate change or the spread of invasive or weed species. Migratory waterbirds (including gulls, Laridae) are of particular importance because they perform long-distance movements and use anthropogenic habitats affected by global change [12,13].

Given that dispersal syndromes fail to explain what seeds are ingested and egested by waterbirds, as demonstrated by faecal sampling [14], a key question is how plant traits determine how many seeds survive gut passage, what their retention time is (a predictor of dispersal distance), and what their germination response is after egestion. Experiments feeding seeds to birds in captivity can address such questions, and there have been several studies with ducks and geese (Anatidae) [15,16,17]. However, to date, no experiments have compared the response to gut passage of seeds from different classical dispersal syndromes, even though waterbirds sometimes feed on +fleshy- and dry fruits at the same time [18]. Given the overwhelming emphasis in the literature on endozoochory on fleshy fruited plants, it may be hypothesised that the seeds of such plants are better adapted to survive gut passage than those with dry fruits. Among dry-fruited plants, there is evidence from some experiments that smaller seeds have higher seed survival but shorter retention times than larger seeds, although other studies have found contradictory results [15,18,19].

The lesser black-backed gull *Larus fuscus,* Linnaeus 1758 (LBBG), is a long-distance migratory bird that has increased in numbers across Europe during the last century [20,21] related to the proliferation of landfills and other human-modified habitats. The LBBG is an opportunistic bird with an omnivorous diet connecting anthropogenic and natural habitats at the landscape scale [22]. Recent studies have shown that wintering LBBG disperse seeds of more than 15 plant species growing in the largest rice field area of Spain by endozoochory, mainly by secondary dispersal of seeds attached to their main prey, the alien red swamp crayfish *Procambarus clarkii* [12,23]. Most of these plants are terrestrial weeds that lack fleshy-fruits, and during the winter, LBBG can disperse them over distances of up to 100 km or more, even outside migration periods. The dispersal of pest species is one of the main ecological disservices provided by birds with important ecological and economic costs, but remains understudied [15,17].

Here, we assessed the role of LBBG as seed vectors by endozoochory by a controlled experiment in captivity. We used five weed species previously recorded in LBBG faeces and pellets from rice fields, only one of shows endozoochory syndrome [12,24]. Our objectives were to quantify (1) the proportion of seeds of each species that survived gut passage by the LBBG, (2) the seed retention time, and (3) the germinability and germination time after gut passage compared to control seeds. Based on the available literature, the following hypotheses were tested: after gut passage, (1) seeds of the fleshy-fruited species would have the highest seed survival rate, and an increased germinability and faster germination compared to control seeds, consistent with better adaptation for endozoochory than for dry-fruited species; (2) seeds of dry-fruited species would show reduced germinability and slower germination compared to control seeds; (3) species with smaller seeds would have higher seed survival, but shorter retention time that those with larger seeds.

## 2. Results

### 2.1. Survival

Intact seeds of the five plant species were recovered from the droppings of gulls (Table 1), but seed survival differed significantly between plant species (GLMM1.1, fixed-factor plant species: χ^2^ = 57.72, df = 4, *p* < 0.001). All taxa had an average seed survival of 72–88%, with *Polypogon monspeliensis* having the highest and *Juncus bufonius* the lowest (Table 1). Pairwise differences between species in seed survival were significant, except those between *Cyperus difformis* and *J. bufonius* or *P. monspeliensis*, or between *Amaranthus retroflexus* and *J. bufonius* (Table 2).

**Figure 1:**
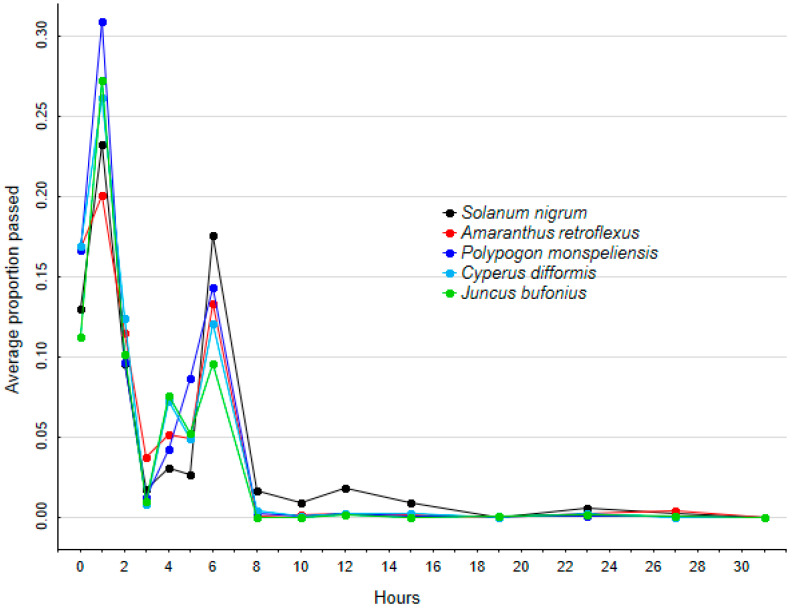
Average proportions of intact seeds recovered from gull faeces, at each sampling time for each plant species.

For those seeds that survived gut passage, the relative proportion of seeds egested at different retention times varied significantly among species (GLMM1.1, random slope retention time—species: χ^2^ = 61.03, df = 1, *p* < 0.001, Figure 1).

### 2.2. Retention Time

Modal retention time was 1 h for all five plant species (Figure 1), whilst means ranged between 2.5 and 3.6 h, with maxima exceeding 23 h (Table 1). Average retention time was highly correlated with seed size (Figure 1, Table 1, Pearson’s correlation between average retention time against average seed mass: r^2^ = 0.95, N = 5, *p* = 0.004), with *S. nigrum* showing the highest value and *J. bufonius* the lowest. *C. difformis* showed a maximum retention time of 23.5 h, while the other four species reached a maximum retention time of 27.5 h (Table 1, Figure 1). Retention time differed significantly between the five plant species (GLMM2, plant species: χ^2^ = 102.64, df = 1, *p* < 0.001). Post hoc tests showed that retention time of *S. nigrum* differed significantly from that of all other plant species (Table 3).

### 2.3. Germinability

**Figure 2:**
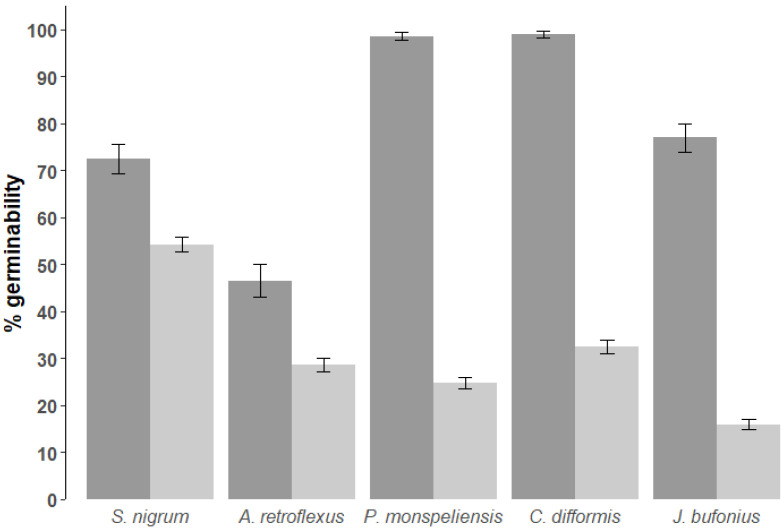
Differences in proportion of germinated seeds between controls (dark grey) and after gut passage (light grey), in order of decreasing seed size from left to right (*Solanum nigrum, Amaranthus retroflexus, Polypogon monspeliensis, Cyperus difformis and Juncus bufonius*). Bars represent 95% confidence intervals. All seeds were stratified before the experiment.

**Figure 3:**
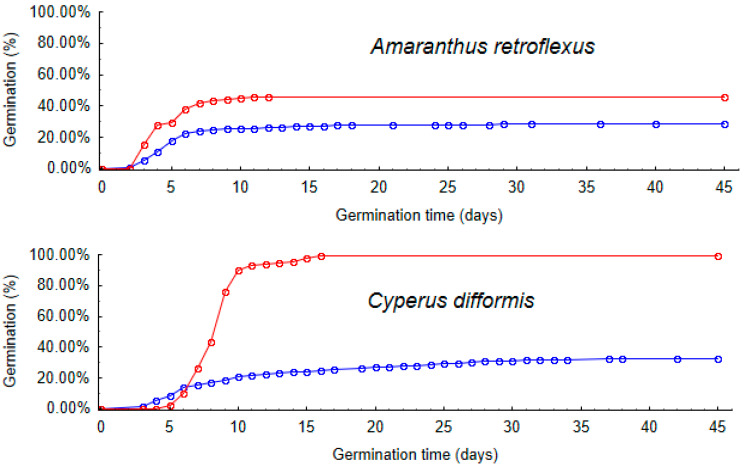

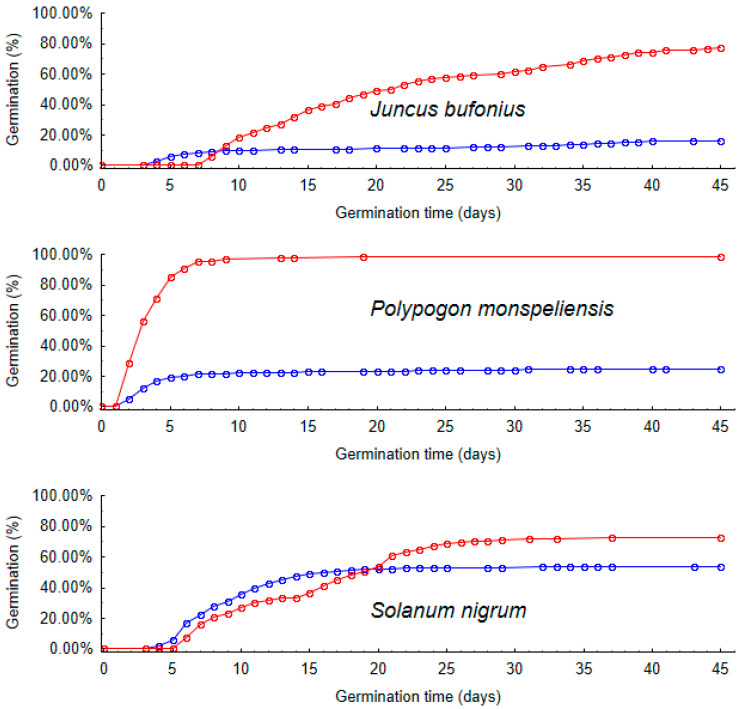
Accumulative percentage curve of germinated seeds of five plant species after gut passage (blue) and for control seeds (red).

After gut passage, *S. nigrum* had the highest germinability (54%), and *J. bufonius* the lowest (16%, Table 4, Figure 2 and Figure 3). In contrast, for control seeds, germinability was >98% for *C. difformis* and *P. monspeliensis*, and was lowest for *A. retroflexus* (46.5%).

The effect of treatment on germinability differed significantly between plant species (GLMM3.1, interaction plant species * treatment: χ^2^ = 294.71, df = 1, *p* < 0.001, Figure 2). When the effect of treatment was explored separately for each plant species, germinability was significantly lower after passage than for control seeds for all species (GLMM3.2s, Table 4, Figure 2 and Figure 3). However, the difference between controls and gut passage was related to seed size, with the significant loss of germinability after passage decreasing in larger seeds (Figure 1 and Figure 2, Pearson’s correlation between germination ratio between treatment and control seed against average seed mass: r^2^ = 0.82, N = 5, *p* = 0.035).

### 2.4. Germination Time

For both control seeds and after gut passage, seeds of *J. bufonius* (the species with the smallest seeds) had the longest germination time, and *P. monspeliensis* had the shortest (Table 5). There was no relation to seed size, since the largest *S. nigrum* seeds also had relatively long germination times (Table 5).

The change in germination time between control and passage seeds differed significantly between plant species (GLMM4.1, random interaction plant species * treatment: χ^2^ = 584.01, df = 4, *p* < 0.001, Figure 3). While experimental seeds of *A. retroflexus*, *C. difformis*, and *P. monspeliensis* showed a significant increase in germination time after passage, seeds of *J. bufonius* and *S. nigrum* (i.e., the smallest and largest seeds) experienced a significant reduction in germination time after gut passage (Table 6, Figure 3).

## 3. Discussion

This study advances our understanding of endozoochory of seeds by non-frugivorous birds, providing information on parameters influencing effective dispersal, such as seed survival, retention time and germinability. It is the first experimental study of gulls (Laridae) to address their important role as vectors of dry-fruited plants [25]. Like other waterbirds such as ducks, geese and storks, gulls are important vectors of weeds in rice fields and other agricultural habitats [13,26]. Our research contributes to the understanding of mechanisms of dispersal of weeds and invasive plants, most of which have been assigned to abiotic dispersal syndromes and have been assumed to rely on humans for long-distance dispersal [17,27].

### 3.1. Retrieval, Viability and Retention Time of Seeds in Relation to Plant Traits

We found that the five weed species (four dry fruited and one fleshy fruited) had high rates of seed survival after gut passage, exceeding 72%. Similar experiments with mallards *Anas platyrhynchos* and other Anatidae have found some plant species to have much lower rates of seed survival [16,18]. We may even have underestimated seed survival since our experiment ended after 33 h, and some seeds have been recovered after longer periods from Anatidae [19]. Species with the highest seed survival were dry fruited and of intermediate size (*P. monspeliensis* and *C. difformis*). In experiments with Anatidae, smaller seeds generally showed higher survival because they escape from the gizzard into the intestines more quickly, although other characteristics such as hardness, seed shape and phylogeny were also important predictors of seed survival [16], see also [28,29].

Longer retention times may benefit plants by increasing seed dispersal distances, making it more likely that they can reach and colonize previously unoccupied habitat patches. Given the generally high rates of seed survival, our finding that average retention times were strongly related to seed size was to be expected, with larger seeds taking longer to pass into the intestines and then be egested. In Anatidae experiments where species with larger seeds were found to have shorter retention times, this is likely because these species had low seed survival, and the only seeds to be egested were the fraction that passed quickly into the intestines [15,18]. We compared seeds from fleshy-fruited and dry-fruited plants fed simultaneously to birds, and found that seed size, rather than fruit fleshiness, is the main determinant of retention time. However, when comparing separate experiments, the retention times of seeds are generally much lower for frugivorous than for non-frugivorous birds, as a consequence of the effects of fruit pulp on gut activity [30]. This suggests that non-frugivorous vectors have a greater potential for long-distance seed dispersal than frugivorous ones.

If we had not removed the pulp from *S. nigrum* seeds prior to our experiments, we may have found different results, with a change in how this species compared with dry-fruited ones. However, our interest was in comparing how the seeds per se compared in their response to gut passage. We found no evidence that, apart from being the largest of the five species, there was anything special about *S. nigrum* seeds and their gut passage in comparison with the other species assigned to barochory or epizoochory syndromes. This is consistent with Costea et al. [31], who found that there is a diversity of seed architectures amongst angiosperms that allow different species to survive gut passage, and that there is no evidence for a fundamental difference between seeds from fleshy- or dry-fruited species. When combined with the growing body of field data showing how endozoochory is a frequent and often predictable dispersal mechanism for many species assigned to other dispersal syndromes [9], our results provide further evidence that seeds of dry-fruited species can have adaptations (or exaptations) for non-classical endozoochory. Species such as *A. retroflexus* and *J. bufonius* have been repeatedly shown to disperse by endozoochory in field studies of gulls, Anatidae or other waterbirds [12,32].

Germination experiments confirmed the viability of the egested seeds, although we found germinability to be higher in control seeds in all five species. This result may have changed if the seeds had not been kept at cold temperatures between collection and our experiments, since this stratification may have broken dormancy in control seeds. If the seeds had been kept at ambient temperature, gut passage may have increased germinability compared to control seeds, as observed in some previous studies [19,33]. This is supported by a separate finding that storage time between faeces egestion and seed extraction was correlated with germinability (Figure S1), although this result was confounded with retention time (see Methods). With hindsight, we should have processed faecal samples in a random order with respect to retention time.

We did not expect to find the observed positive correlation between seed size and the germinability of egested seeds relative to control seeds, especially as larger seeds had longer average retention times, which would be expected to translate into reduced viability [19,33]. The impact of gut passage on germination time was unrelated to seed size, since the smallest and largest seeds (of *J. bufonius* and *S. nigrum*) were the only two for which seeds germinated faster compared to the controls. *J. bufonius* is easily the most abundant seed in LBBG faeces and pellets from rice fields of the Guadalquivir estuary [12,24], and its rapid germination after gut passage may promote endozoochory given intense intraspecific competition for this weed, which is highly abundant in rice fields, especially between harvest and sowing. It has smaller seeds than the other weeds we studied, but this allows production of more seeds than other species [34,35], with 34,000 seeds/ramet [36].

The relationship between seed size and germinability or germination time is clearly complex and confounded by other seed traits such as dormancy strategies. Kleyheeg et al. 2018 [37] tested 30 wetland plant species and found that germinability increased with seed size for control seeds, but this relationship was less pronounced after artificial digestion. They did not find a significant influence of seed size on time to germination. In contrast, we found that size was more related to germinability *after* gut passage than *before* (Figure 3), although this difference was not significant. Some experimental studies have found a negative correlation between seed size and germinability within plant species [29].

Our experimental estimations of seed survival and retention time might differ from real values in natural conditions. Avian diet can have an important influence on these parameters [38], although our birds were fed on an animal diet, which reflects their preference for crayfish in the field. Captivity can change seed survival and retention time due to the limited mobility of the birds compared with those active in the field. Kleyheeg, et al. [39] found that seed survival increased significantly with forced activity in ducks. LBBGs spend long periods of the day inactive, roosting in rice fields or neighbouring habitats [40], so our experiment may reflect field conditions quite well.

Martín-Vélez et al. [13] developed a spatial seed dispersal model based on GPS data for LBBGs and for field and experimental data for four weed species used in our study (*J. bufonius*, *C. difformis*, *P. monspeliensis* and *A. retroflexus*). These authors estimated median endozoochory dispersal distances of 690 to 940 m, with the maximum exceeding 150 km. Their model found that seeds would be deposited in suitable habitats for weed establishment such as other rice fields, natural wetlands and other irrigated crops. These geographic distance estimations are longer than those calculated for abiotic dispersal mechanisms such as wind [41]. For example, the maximum dispersal distance estimated for *J. bufonius* via wind was 100 m [42].

### 3.2. Characteristics of LBBG That Make Them Good Seed Dispersal Vectors

The LBBG is a long-distance migrant with a broad flyway, moving from North Europe to northwest Africa [43,44]. It typically combines migration with foraging during frequent and long-migratory stopovers, the so-called fly-and-forage migration strategy [44,45,46]. Although it does not achieve long daily distances compared with other migratory birds [average instantaneous speed of 38.6 km/h; 44 km/day in autumn and 98 km/day in spring; 44] (average instantaneous speed of 38.6 km/h; 44 km/day in autumn and 98 km/day in spring), the LBBG is still faster and can achieve longer daily distances than other fly-and-forage migrants such as raptors [45]. On the other hand, LBBG have the ability to find suitable habitats for feeding nearly everywhere along their migratory route due to their opportunistic feeding in inland as well as marine habitats, increasing the possibility that seeds are egested in favourable habitats [22]. The LBBG may thus be a good vector for long-distance dispersal of weeds, aliens and other plants in a step-by-step manner. Throughout the winter, the LBBG performs daily movements between feeding and roosting sites of up to 80 km [22] favouring dispersal of seeds from highly anthropized habitats to more natural environments. Due to their common use of landfills, agricultural fields and other human-transformed habitats, the LBBG may be a particularly suitable vector for weeds and invasive species (this study; [22]).

The LBBG is an opportunistic feeder that includes many different food items in its diet [47,48]. This omnivorous diet may enable higher seed survival compared to granivorous bird species. Moreover, the structure of the gizzard, responsible for mechanical digestion, plays a key role in seed survival [28]. In the case of ducks specialized in the digestion of seeds, the gizzard is very thick and muscular, with the presence of grit which improves mechanical digestion of seeds, although grit may also increase germination rate through scarification [49]. Omnivorous birds such as LBBGs have relatively small gizzards compared to herbivorous or granivorous birds, and are thus more likely to allow seeds to survive gut passage [37]. This is likely to explain the particularly high recovery rate of intact seeds in our experiment. Overall, gulls can be expected to provide effective seed dispersal with relatively high-quantity and quality components [50], although further research into the survivorship of dispersed seeds and resulting seedlings is required.

### 3.3. Ecological and Management Implications of Weed Dispersal by LBBG

All five species we studied are noxious agricultural weeds in Europe and other parts of the world, causing important losses to food production globally [51]. Some of them are also invasive aliens and outcompete native flora. *P. monspeliensis*, *A. retroflexus* and *C. difformis* in particular have evolved under the use of intense herbicide treatments, developing herbicide resistance that results in significant economic costs [52,53]. In India, *P. monspeliensis* is one of the main weeds of wheat and rice fields [54], and is spreading rapidly in Spain [55]. It has invaded saltmarshes in Southern California, outcompeting native *Salicornia virginica* [56] and leading to important restoration efforts [57]. *Amaranthus* species are among the most serious agricultural weeds in the world [58,59]. Their life history traits and allelopathic chemicals allow them to outcompete other plants and significantly reduce grain production and quality. Both *A. retroflexus* and *S. nigrum* are well known for their allelopathic effects [60,61] which significantly reduce germination and growth of agricultural crops by toxic chemical production. Some weed species from our study have also been associated with livestock poisoning, e.g., via bacterial infection, such as *Clavibacter toxicus* of seedheads in *P. monspeliensis*, such as *Clavibacter toxicus* of seedheads in *P. monspeliensis* [62] or chemical compounds in *A. retroflexus* [63,64]. *Solanum nigrum*, native to Eurasia and invasive in Australia, is a spill over host of Tobamoviruses, which are amongst the most damaging viruses to horticultural crops [65].

*C. difformis* is one of the world’s worst weed species [66], particularly in rice-growing regions of Europe, US and Asia [67]. In the Guadalquivir estuary, the main rice cultivation area in Spain, it is considered the worst weed, with resistance to the most commonly used herbicides. On the other hand, the globally distributed *J. bufonius* is considered invasive in the US and has been recently recorded in Antarctica, where it may spread rapidly due to climate warming [68]. Typically, the potential introduction vectors reported by Cuba-Díaz et al. [68] in Antarctica were humans (tourists, scientists, etc.), with no mention of birds. Our results suggest that gulls and other migratory birds using Antarctica may have a key role transporting propagules of new species that may thrive under new, warmer conditions.

Most studies of weed science during the last two decades have focused on herbicide research. However, the 21st century requires a holistic approach to weed science, with more contributions to weed biology, ecology and genetics [59,69]. Understanding the spread of weeds by wildlife is particularly important. Knowledge about dispersal of weeds by birds may be an important contribution to the challenge of achieving weed control through a multidisciplinary perspective. For example, identifying the season when avian vectors are more abundant may facilitate effective integrated weed management and identify optimal timing for weed control. Ricefields in Spain and other parts of the Mediterranean region are major wintering sites for many species of waterbirds dispersing weeds, such as gulls, storks, geese, and other waterbirds [20]. Proactive management activities in the winter could contribute to an integrated approach to weed management in rice fields. On the other hand, waterbirds reduce the density of weed seeds in rice fields through their foraging [20].

Integrating genetic and genomic studies of weeds transported by birds and their herbicide resistance would be another important future field of research. Even a single herbicide-resistant weed seed may become enough to promote the colonization of a whole field [70]. Globally, the role of waterbirds in the dispersal dynamics of weeds has been ignored to date, and recognizing it may help to predict their spread and to develop suitable management strategies for control.

## 4. Materials and Methods

### 4.1. Plant Species and Seed Collection

**Figure 4:**
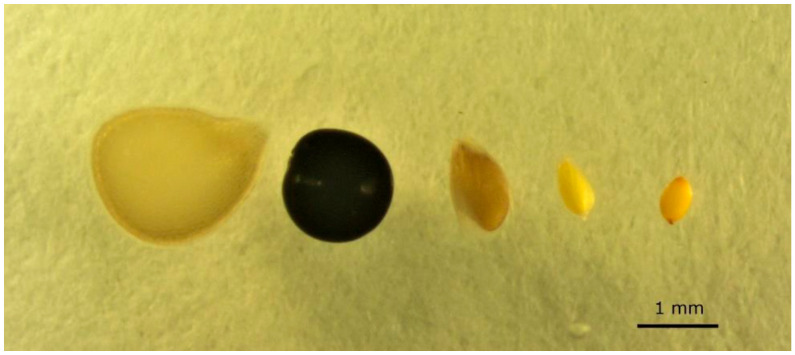
Seeds of the five weed species fed to lesser black-backed gulls, in order of decreasing seed size. From the left to the right: *Solanum nigrum*, *Amaranthus retroflexus*, *Polypogon monspeliensis*, *Cyperus difformis* and *Juncus bufonius*. Dispersal syndromes according to [75] are: *S. nigrum* endozoochory, *A. retroflexus* barochory, and the other three epizoochory.

Five species of rice field weeds, with different dispersal syndromes, previously recorded in LBBG droppings from Spanish rice fields were selected (Figure 4) [12,13,23]. *Solanum nigrum* (black nightshade) and *P. monspeliensis* (annual beard grass) are European species introduced elsewhere, such as America and Oceania [71,72]. *Cyperus difformis* (rice sedge) is native to tropical and subtropical regions and widely distributed in southern Europe, Asia, America, Africa, and the Pacific islands [73]. *Amaranthus retroflexus* (red-rooted pigweed) is native to the Americas and is invasive throughout the world [51]. *Juncus bufonius* (toad rush) is cosmopolitan, abundant in crops throughout Europe, and known to be dispersed by waterbirds in a variety of habitats and locations [9,24,74]. It was easily the most abundant taxon of all seeds recovered from LBBG egesta in the field.

Seeds of *A. retroflexus*, *S. nigrum* and *P. monspeliensis* were collected in spring (March and April) 2018 and those of *C. difformis* in September 2018, from rice fields in Isla Mayor (37°07.93′ N, 006°09.82′ W, Seville, Spain), in the Guadalquivir River delta. *Juncus bufonius* seeds were collected in September 2018 in swampy areas of Hungary near crop fields (47.273410 N, 21.40819 E, Berettyóújfalu, Hungary).

In the laboratory, seeds were separated, cleaned, dried and stored in plastic vials at 5 °C in the fridge (in the dark) until the experiments started. In the case of *S. nigrum* seeds, the pulp of the fleshy fruit was previously removed by placing the fruits in between two filter papers, squeezing them with a flat spoon, then letting them dry. Half of all seeds were used for the gut passage experiment, while the other half were kept as control seeds for germination tests.

### 4.2. Gull Captures

LBBG is the second-most abundant wintering species in Andalusia (67,365 birds in 2022) [76], with over 10,000 individuals concentrating in the rice fields of the Guadalquivir estuary [13]. Seven LBBG individuals were captured between the 14th and 21st January 2020 at the Urban Solid Waste Selection and Transfer Station at Bollullos de la Mitación (37°19.94′ N, 006°7.88′ W, Seville, Spain). Gulls were captured with mist nets (13 m × 2.40 m and 2 × 2 cm mesh size), suitable for the selective capture of large birds. After capture, the gulls were banded with unique numeric rings, placed in individual cardboard boxes (approved by the Ministry of Agriculture, Fisheries and Environment and provided by Environmental Regional Agency, Junta de Andalucía) and transported to the Endangered Species Recovery Centre (CREA) “Dunas de San Antón” (36°35.41 N, 006°14.57 W, El Puerto de Santa Maria, Cadiz, Spain).

### 4.3. Feeding Trial

The experimental design and procedures carried out at CREA aimed to minimize stress for the gulls. Birds were acclimatized to a cage of 2.70 m × 3.80 m × 2 m with a 2 × 2 cm metal mesh, located in the open air under natural light and temperature. Winters in Southwest Spain are usually mild, with monthly averages rarely below 12 °C. The seven gulls were kept together for a week, allowing visual contact between individuals. Birds were fed ad libitum with fresh fish.

For the experiment, gulls were isolated in individual outdoor cages of 80 cm × 70 cm × 90 cm with a 2 × 2 cm metal mesh. The cages were covered with green 99% concealment shading mesh that reduced the stress of the birds. Laboratory filter papers were placed on the floor of the cages to collect faeces and any regurgitates during the experiment. Water and fresh fish were offered ad libitum throughout the experiment.

On 27–28 January 2020, each gull was force fed with 1000 seeds (200 of each species) embedded in a 2 cm diameter bread ball to facilitate ingestion. A small amount of water (0.5 mL) was administrated with a syringe, facilitating swallowing and preventing regurgitation. The droppings were collected at regular intervals: after 30 min of the beginning of the experiment, every hour for the next 5 h, every 2 h for the next 8 h, and every 4 h for the next 20 h until the experiment’s completion (a total of 33 h and 30 min). Laboratory filter papers with droppings were placed in hermetically sealed plastic zip bags and stored in a refrigerator at 5 °C until processing (between 22 and 73 days later). As soon as the experiment finished, the birds were released. Gulls were placed into individual cardboard boxes and released in the Cetina Saltpan (36°33.98′ N, 006°9.04′ W, Puerto Real, Cadiz, Spain), a roosting area for this species. All birds flew away without any sign of weakness or damage.

### 4.4. Seed Retrieval and Germination Experiment

Gull droppings were processed between 19 February and 10 April 2020. Seeds were extracted by filtering the droppings through a 200 mm-diameter sieve with 45 µm mesh size. The filtrate from each individual faecal sample was transferred to a Petri dish, and inspected under a Zeiss SteREO Discovery V8 stereomicroscope. Intact seeds of different plant species were counted and extracted with soft laboratory forceps. Seeds showing any evidence of external physical damage were counted and discarded. Intact seeds were individually placed in 24-well plates with moistened laboratory filter paper at the bottom of the well. Control seeds (200 of each species) were placed in similar plates. Control and experimental seeds were incubated for 45 days in a climatic chamber (Fitoclima 10,000 EHF, ARALAB) equipped with Sylvania T8 Luxline Plus F58W 840 fluorescent tubes, a 12-12 h photoperiod, and temperature cycles of 22 °C (day) and 18 °C (night). Seeds were watered regularly and checked every two days with a Zuzi series 240 binocular stereo microscope, counting and removing germinated seeds. A seed was considered germinated when the radicle emerged 2 mm or more above the seed cover. After 45 days, ungerminated seeds were counted and the germination tests were concluded (on 31 May 2020).

### 4.5. Statistical Analyses

We used general linear mixed-effect models (GLMM) to test how the temporal dynamics of seed ingestion, survival and germinability varied among different plant species. When analysing only egested seeds, we used a random factor to control for variation between bird individuals, which was expected to be important [77]. When comparing control and egested seeds in other analyses, this was not possible because control seeds were not assigned to particular individuals.

### 4.6. Seed Survival

For seeds that survived gut passage, the proportion of all seeds egested for a given species recovered in a dropping at a given time interval was used as the response variable in GLMM1, following a binomial distribution. Plant species was included as a fixed factor and gull identity as a random factor. The model also included a random slope term for each plant species at each time of sample collection.

### 4.7. Retention Time

The time elapsed between the ingestion of the seeds and their egestion was calculated as the mean value of each time interval in which the seed was egested (for example, if a seed was egested between 7.5 h and 9.5 h, a retention time of 8.5 h was considered). Logarithmic transformations were performed to normalize the response variable. We analysed this response variable in GLMM2 with a Gaussian error distribution and a log-link function, where the plant species was included as a fixed factor and gull identity as a random factor.

### 4.8. Germinability

We analysed germinability (i.e., the probability that a seed germinates) as a binomially distributed response variable in different sets of GLMMs. In GLMM3.1, we explored the effect on germinability of treatment (gut passage or control) and plant species as fixed factors, as well as the plant species × treatment interaction. We further explored treatment effects for each plant species in GLMM3.2, with treatment as a fixed factor.

Because faecal samples were processed in the order in which they were recovered, storage time (interval in days between recovering faeces and placing seeds for germination tests) was strongly correlated with retention time (r between storage time and log-transformed retention time was 0.10 to 0.30 for each plant species, *p* < 0.001, Supplementary Figure S1). Both storage time and retention time can strongly affect germinability [11,18], and in our case, with such strong correlations, we could not satisfactorily separate their effects. Storage time was strongly negatively correlated with germinability in our experiment, and had strong correlations (four negative and one positive) with germination time (Supplementary Table S1). However, we cannot be sure of the relative roles of storage and retention time in these relationships. For this reason, we did not attempt to analyse the effects of retention time on germinability or germination time in more detail.

### 4.9. Germination Time

Germination time was the number of days that a seed took to germinate. This response variable followed a Poisson distribution and was included in the GLMM with the log link function. Following a similar approach to germinability, a first model (GLMM4.1) included treatment (control or gut passage) and plant species as fixed factors, and their interaction. We further explored treatment effects for each plant species in GLMM4.2, with treatment as a fixed factor.

GLMMs were performed in the R environment 4.0.3 [78], using function glmmTMB in package glmmTMB [79]. Package emmeans were used for post-hoc analyses [80] and ggplot2 for figure preparation [81].

## 5. Conclusions

Five species of weeds of global concern have high potential for endozoochory via gulls, with high rates of seed survival and germinability after gut passage. Dispersal syndromes based on morphological fruit traits cannot adequately predict how weeds disperse, and seeds of fleshy-fruited weeds are not better adapted for gut passage than those of dry-fruited weeds. Endozoochory rates are more strongly linked to other traits such as seed size. Our study highlights the important, and previously underestimated, role of migratory waterbirds as vectors for weeds, enabling a high rate of dispersal, which is itself a trait of “weediness”. Similar feeding experiments are needed to further understand the role of non-classical endozoochory in the dispersal of other weed species and the role of other waterbird vectors known to consume weeds (such as storks and shorebirds) [11,12,24], and other non-frugivorous birds such as game birds and passerines [9]. Non-classical endozoochory by birds is an overlooked mechanism in weed expansion and management research.

## Figures and Tables

**Table 1:** (a) Percentage of seeds of each plant species that survived gut passage through seven lesser black-backed gulls. (b) Average retention time (in hours) for seven gulls. S.D.: Standard deviation, Min: minimum, Max: maximum.

	(a) Survival Rate					(b) Retention Time				
Plant Species	Average	S.D.	Min.	Max.	Median	Average	S.D.	Min.	Max.	Median
*Amaranthus retroflexus*	76.93	6.95	64.00	85.00	78.50	2.80	3.22	0.25	27.5	2.00
*Cyperus difformis*	81.86	9.01	69.00	92.00	86.00	2.53	2.66	0.25	23.5	1.00
*Juncus bufonius*	72.43	9.48	57.50	83.00	72.00	2.47	2.45	0.25	27.5	1.00
*Polypogon monspeliensis*	87.71	6.38	80.50	96.00	85.00	2.58	2.62	0.25	27.5	1.00
*Solanum nigrum*	76.71	21.97	31.50	100.00	80.00	3.55	4.00	0.25	27.5	2.00

**Table 2:** Tukey HSD post-hoc test showing pairwise differences between plant species (*Solanum nigrum, Amaranthus retroflexus, Polypogon monspeliensis, Cyperus difformis and Juncus bufonius*) in the proportion of seeds recovered in gull faeces. Significant *p*-values are highlighted in bold.

Contrast	Estimate	SE	t. Ratio	*p* Value
*A. retroflexus*—*C. difformis*	−0.18	0.06	−2.86	**0.036**
*A. retroflexus*—*J. bufonius*	−0.05	0.06	−0.73	0.949
*A. retroflexus*—*P. monspeliensis*	−0.23	0.06	−3.68	**0.002**
*A. retroflexus*—*S. nigrum*	0.20	0.06	3.18	**0.014**
*C. difformis*—*J. bufonius*	0.13	0.06	2.09	0.228
*C. difformis*—*P. monspeliensis*	−0.05	0.06	−0.79	0.932
*C. difformis*—*S. nigrum*	0.38	0.06	6.00	**<0.0001**
*J. bufonius*—*P. monspeliensis*	−0.18	0.06	−2.88	**0.034**
*J. bufonius*—*S. nigrum*	0.25	0.07	3.81	**0.002**
*P. monspeliensis*—*S. nigrum*	0.43	0.06	6.86	**<0.0001**

**Table 3:** Tukey HSD post-hoc test showing pairwise differences between plant species (*Solanum nigrum, Amaranthus retroflexus, Polypogon monspeliensis, Cyperus difformis and Juncus bufonius*) in the retention time of the seeds recovered in faecal samples of lesser black-backed gulls. Significant *p*-values are highlighted in bold.

Contrast	Estimate	SE	t. Ratio	*p* Value
*A. retroflexus*— *C. difformis*	0.03	0.01	2.47	0.097
*A. retroflexus*— *J. bufonius*	0.04	0.01	2.62	0.067
*A. retroflexus*—*P. monspeliensis*	0.03	0.01	2.52	0.087
*A. retroflexus*— *S. nigrum*	−0.08	0.01	−5.92	**<0.001**
*C. difformis*—*J. bufonius*	<0.01	0.01	0.24	0.999
*C. difformis*—*P. monspeliensis*	<0.01	0.01	−0.01	0.999
*C. difformis*—*S. nigrum*	−0.11	0.01	−8.40	**<0.001**
*J. bufonius*—*P. monspeliensis*	<0.01	0.01	−0.25	0.999
*J. bufonius*—*S. nigrum*	−0.12	0.01	−8.30	**<0.001**
*P. monspeliensis*—*S. nigrum*	−0.11	0.01	−8.57	**<0.001**

**Table 4:** Differences in germinability (percentage of seeds) after gut passage compared with control seeds. S.D.: Standard deviation. C.I.: 95% Confidence Interval.

	Experimental Seeds					Control Seeds				
	N	Average	SD	CI		N	Average	SD	CI	
*Amaranthus retroflexus*	1077	28.69	45.26	25.99	31.40	200	46.50	50.00	39.53	53.47
*Cyperus difformis*	1146	32.55	46.88	29.83	35.26	200	99.00	9.97	97.61	100.39
*Juncus bufonius*	1014	15.98	36.66	13.72	18.24	200	77.00	42.19	71.12	82.88
*Polypogon monspeliensis*	1210	24.79	43.20	22.36	27.23	200	98.50	12.18	96.80	100.20
*Solanum nigrum*	1070	54.21	49.85	51.22	57.20	200	72.50	44.76	66.26	78.74

**Table 5:** Germination time (in days) of five plant species after passage through guts of seven LBBG, and for control seeds. A.: Average; M.: Median; S.D.: Standard deviation. C.I.: 95% Confidence Interval.

	Passage Seeds								Control Seeds							
	N	A	M	Max	Min	SD	CI		N	A	M	Max	Min	SD	CI	
*Amaranthus retroflexus*	309	6.51	5	2	40	5.55	5.15	6.03	92	4.82	4	3	12	2.02	1.77	2.36
*Cyperus difformis*	373	11.66	8	3	42	8.73	8.15	9.41	198	8.70	9	5	16	1.98	1.80	2.20
*Juncus bufonius*	162	14.83	7	4	43	13.00	11.73	14.60	154	19.55	16	8	45	10.21	9.18	11.50
*Polypogon monspeliensis*	300	5.76	4	1	41	6.76	6.26	7.35	197	3.82	3	2	19	2.24	2.04	2.49
*Solanum nigrum*	579	9.79	8	3	43	5.26	4.98	5.58	145	14.84	15	6	37	6.97	6.25	7.88

**Table 6:** Differences in germination time between control seeds and seeds after gut passage through seven gulls.

	Control		Passage					
Plant Species	Average	SE	Average	SE	Estimate	SE	Chisq	*p*
*Amaranthus retroflexus*	4.82	0.21	6.51	0.32	0.30	0.05	35.21	<0.001
*Cyperus difformis*	8.70	0.14	11.66	0.45	0.29	0.03	110.48	<0.001
*Juncus bufonius*	19.55	0.82	14.83	1.02	−0.28	0.03	102.42	<.001
*Polypogon monspeliensis*	3.83	0.16	5.76	0.39	0.41	0.04	92.79	<0.001
*Solanum nigrum*	14.84	0.58	9.79	0.22	−0.42	0.03	253.08	<0.001

## Data Availability

The data presented in this study are openly available in 10.6084/m9.figshare.22093121.

## Supplementary Materials

The following supporting information can be downloaded at: https://www.mdpi.com/article/10.3390/plants12071470/s1, Figure S1: Relationship between storage time and log10-transformed retention time of seeds from five plant species that survived gut passage; Table S1: Effect of storage time on germinability of five plant species that survived gut passage. Significant results in bold.
