# Supplementary material for: Seed Size, Not Dispersal Syndrome, Determines Potential for Spread of Ricefield Weeds by Gulls
Source: Plants (Basel). 2023 Mar 27;12(7):1470. doi: 10.3390/plants12071470 (PMC10096937; doi:10.3390/plants12071470)
Supplement: Supplementary file 1 [file plants-12-01470-s001.zip › plants-2286531-supplementary.pdf]

Table S1. Effect of storage time on germinability of five plant species that survived gut passage. Significant results in bold.

	Germinability				Germination time			
	Estimate	SE	Chisq	P	Estimate	SE	Chisq	P
Amaranthus retroflexus	-0.04	0.004	9.26	**<0.001**	-0.004	0.001	3.33	**<0.001**
Cyperus difformis	-0.02	0.004	5.81	**<0.001**	-0.011	0.009	12.25	**<0.001**
Juncus bufonius	-0.03	0.006	5.60	**<0.001**	-0.012	0.001	6.62	**<0.001**
Polypogon monspeliensis	-0.04	0.004	9.82	**<0.001**	0.025	0.001	17.20	**<0.001**
Solanum nigrum	-0.05	0.004	11.13	**<0.001**	-0.012	0.0008	15.74	**<0.001**

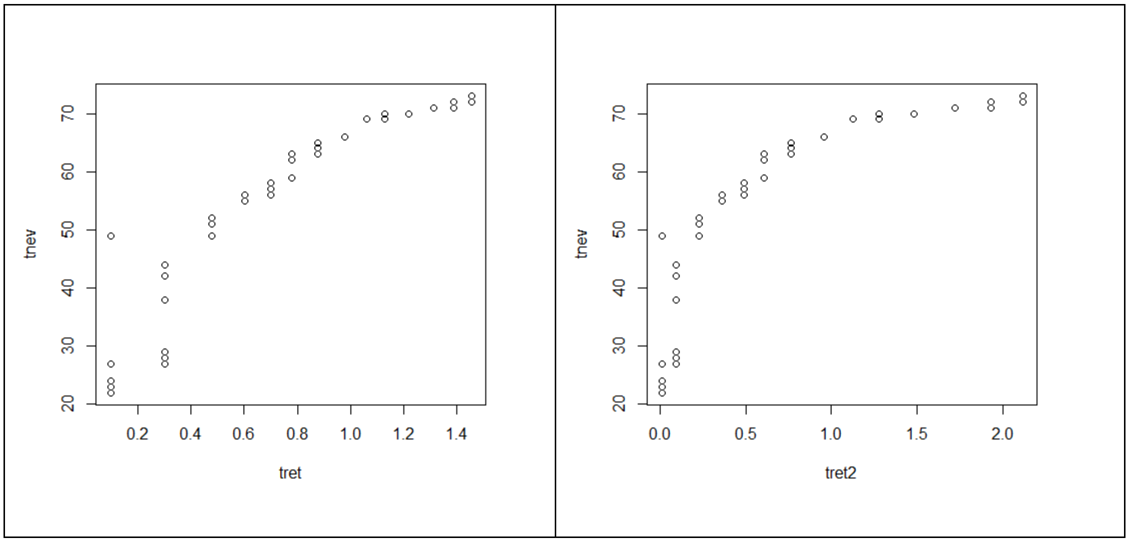

Figure S1. Relationship between storage time and log10-transformed retention time of seeds from five plant species that survived gut passage.
